# Mobile Health Apps for Self-Management of Rheumatic and Musculoskeletal Diseases: Systematic Literature Review

**DOI:** 10.2196/14730

**Published:** 2019-11-26

**Authors:** Aurélie Najm, Laure Gossec, Catherine Weill, David Benoist, Francis Berenbaum, Elena Nikiphorou

**Affiliations:** 1 Department of Rheumatology Nantes University Hospital Nantes France; 2 INSERM UMR 1238 Nantes University of Medicine Nantes France; 3 INSERM UMR S1136 Institut Pierre Louis d'Epidémiologie et de Santé Publique Sorbonne Université Paris France; 4 Rheumatology department Pitié Salpêtrière Hospital AP-HP Paris France; 5 Bibliothèque interuniversitaire de Santé Paris Descartes University Paris France; 6 Department of rheumatology Sorbonne Université, INSERM CRSA Saint-Antoine AP-HP, Saint Antoine Hospital Paris France; 7 Department of Inflammation Biology, School of Immunology and Microbial Sciences Faculty of Life Sciences & Medicine King’s College London Paris France

**Keywords:** mobile health, self-management, arthritis, telemedicine, musculoskeletal diseases

## Abstract

**Background:**

Although the increasing availability of mobile health (mHealth) apps may enable people with rheumatic and musculoskeletal diseases (RMDs) to better self-manage their health, there is a general lack of evidence on ways to ensure appropriate development and evaluation of apps.

**Objective:**

This study aimed to obtain an overview on existing mHealth apps for self-management in patients with RMDs, focusing on content and development methods.

**Methods:**

A search was performed up to December 2017 across 5 databases. For each publication relevant to an app for RMDs, information on the disease, purpose, content, and development strategies was extracted and qualitatively assessed.

**Results:**

Of 562 abstracts, 32 were included in the analysis. Of these 32 abstracts, 11 (34%) referred to an app linked to a connected device. Most of the apps targeted rheumatoid arthritis (11/32, 34%). The top three aspects addressed by the apps were pain (23/32, 71%), fatigue (15/32, 47%), and physical activity (15/32, 47%). The development process of the apps was described in 84% (27/32) of the articles and was of low to moderate quality in most of the cases. Despite most of the articles having been published within the past two years, only 5 apps were still commercially available at the time of our search. Moreover, only very few studies showed improvement of RMD outcome measures.

**Conclusions:**

The development process of most apps was of low or moderate quality in many studies. Owing to the increasing RMD patients’ willingness to use mHealth apps for self-management, optimal standards and quality assurance of new apps are mandatory.

## Introduction

### Background

Mobile health (mHealth) connects patients, their families, and health care professionals by creating a network with mobile and specialized devices with wearable sensors, recording health parameters, and gathering health data. Health information can be subsequently converted and transferred to physicians and other health care professionals involved in the care of patients via medical application interfaces. By enabling patients to access and share their health information, mHealth empowers patients to become more engaged and to take initiative in self-management and shared management of their health.

Since the first description of the concept of mHealth [1], its popularity has exponentially increased. This is primarily because of the fast expanding technological advances including the development of smartphones and fourth generation mobile communication system networks. The impressive popularity of mHealth apps in the last decade is reflected by the number of downloads in recent years, exceeding 200 million in 2010 [2].

On the wider mHealth market, various apps have been developed for different purposes. The latter include apps for disease prevention among healthy users [3] and apps for people with existing chronic health conditions [4]. A recent study, for example, demonstrated that apps can contribute to improve disease control in people with diabetes [5], hypertension [6], or asthma [7] and can help for the monitoring and self-management of obesity [8], mental health diseases [9], and multimordbidities [10]. A total of 17.3 million people report having rheumatic and musculoskeletal diseases (RMDs), the most frequent being low back pain and osteoarthritis (OA) [11]. As RMDs have multidimensional consequences on health, the increasing availability of apps has an important role to play in enabling people with RMDs to better self-manage their health [12]. Moreover, in a recent study of people with rheumatoid arthritis (RA), 86% agreed that an app to support self-management would be useful and welcomed [13]. This being said, the dramatic increase and adoption of mHealth apps and the business it generates raise some fundamental questions, such as (1) How is the scientific content controlled? and (2) How can we make sure that apps are appropriate for patients?

### Objectives

Despite the growing enthusiasm for this topic among physicians and researchers, there is a lack of evidence describing the development and evaluation of apps for people with RMDs that fulfill quality requirements for their implementation as part of routine care. This formed the rationale for this systematic review of literature as part of a larger project to inform points to consider for the development, evaluation, and implementation of mHealth apps for self-management of RMDs. The overarching aim of this systematic review was to obtain a clear view on existing mHealth apps for patients with RMD. Specific objectives were to better characterize (1) the target population of available apps, (2) their purpose and content, and (3) strategies of mHealth app development.

## Methods

### Search Strategy

A systematic literature review (SLR) was performed following the preferred reporting items for systematic reviews and meta-analyses methodology [14]. The search was performed using the Cochrane Library, EMBASE, MEDLINE, PsychINFO, Web of Science, and gray literature (internet and international rheumatology societies’ websites) up to December 2017. Relevant keywords and Medical Subject Headings terms relative to 3 key domains were used: RMDs, self-management, and mHealth (see Multimedia Appendices 1 and 2). The search strategy was developed with support from 2 experienced librarians (DB and CW). The clinical questions and inclusion criteria were predefined according to the population, intervention, control, and outcomes (PICO) statement [15]. PICO is a framework that allows to facilitate literature search and to formulate the scientific questions. The target population was patients with any RMDs. The intervention was the description or use of any apps for self-management, irrespective of whether these apps were connected or not to a device.

Any articles describing the development, evaluation, usability, accessibility, effectiveness, and assessment of patient-reported outcomes (PROs) collected through the internet or through electronic apps, and satisfaction over the use of an app for disease self-management, were included. English language was applied as a limit for the articles. Double screening by 2 independent reviewers (AN and EN) was performed for all abstracts against inclusion/exclusion criteria with agreement of 99% in selected papers. Disagreements were resolved by discussion.

### Data Analysis

Data regarding type of app, target population, country of development, themes and objectives, development process, funding sources, and functionality of the app were collected. The development process was classified into 3 main categories: (1) patients or health care providers involved in both design and evaluation phases, (2) patients or health care providers involved in the evaluation but not in the design process, and (3) neither patients nor health care providers involved in the design or evaluation phases.

The commercial availability of the app was also checked on Google and Apple Store. Owing to the vast heterogeneity of the included studies, a meta-analysis was not considered appropriate. Descriptive statistics were performed using GraphPad Prism (GraphPad Software, San Diego)

## Results

### Systematic Literature Search Output

**Figure 1:**
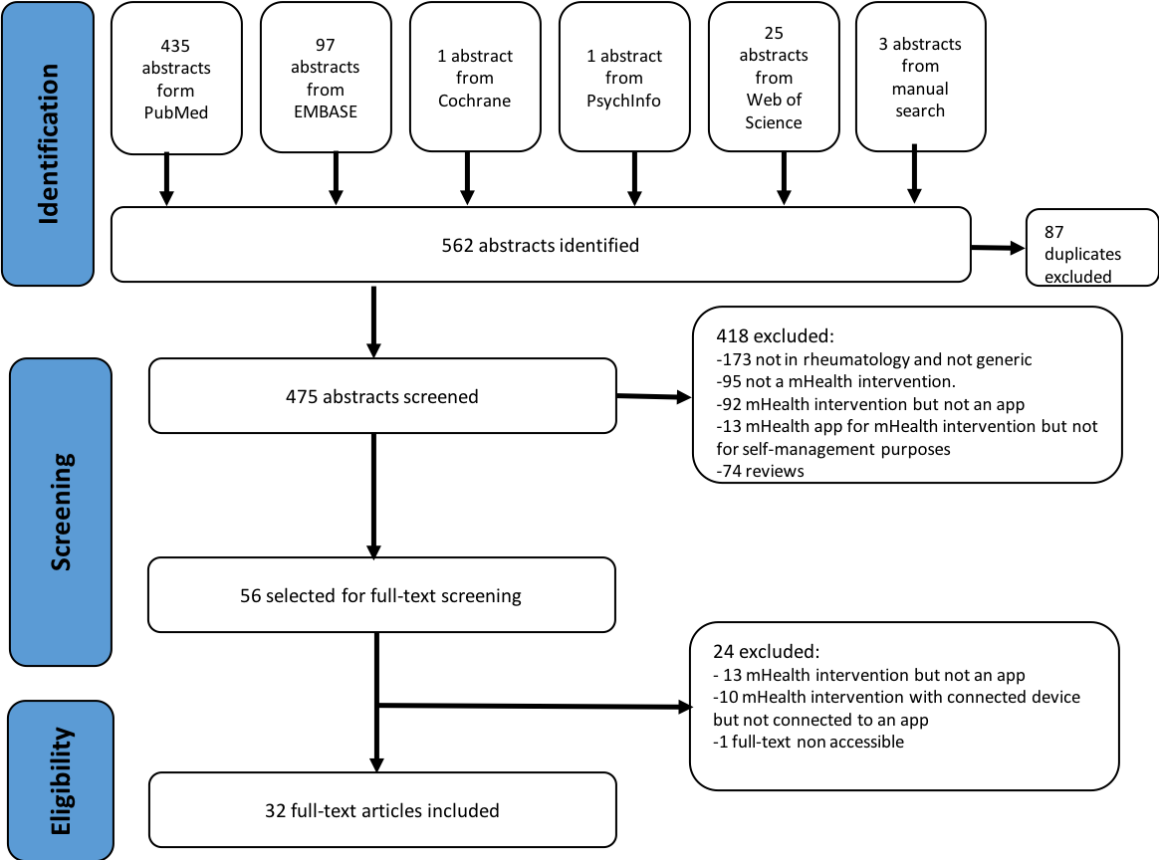
Flow chart summary of the systematic literature review, article identification, screening, and final selection. mHealth: mobile health.

The search identified 562 abstracts. Manual search through screening of national societies and patient associations’ home pages yielded 1 additional reference. After duplicate exclusion, 475 abstracts were screened based on title and abstract. From these, 56 articles were identified as potentially relevant and selected for full-text assessment. After full-text assessment, 32 articles were considered suitable for inclusion in the analysis (Figure 1).

### General Study Characteristics

Out of the 32 included studies, 28 were observational studies and 4 were randomized controlled trials (RCTs). Description of the objective for each study, the target disease, the country of origin, name of the app, and the type of data collected by the app of each RCT [16-21] and observational studies [22-47] is detailed in Multimedia Appendix 3 [48].

### Target Disease of Mobile Health Apps, Country of Origin, and Funding Sources

Out of the 32 included articles, 13 (40%) referred to an app linked to a connected device. Most of the apps (26/32, 81%) were designed for the use of patients living with a specific rheumatic disease, distributed as follows: RA (11/32, 34%), fibromyalgia (5/32, 15%), juvenile idiopathic arthritis (4/32, 12%), OA (3/32, 9%), psoriatic arthritis and ankylosing spondylitis (AS; 1/32, 3%), spine disease (1/32, 3%), and ankle sprain (1/32, 3%). The other apps (6/32, 18%) were either designed for multiple diseases (eg, AS, RA, and systemic lupus erythematosus) or for the general population but used by patients living with RMDs in some studies. The great majority of the apps were developed in the United States (11/32, 34%), Japan (4/32, 12%), Canada (3/32, 9%), and Norway (3/32, 9%).

The funding sources were cited in 93% (30/32) of the articles and reported to be private in 34% (11/32) of the cases. The funding source was reported to be academic in 62% (20/32) of the articles.

### Purposes and Data Collected by Mobile Health Apps

Most of the apps were designed for self-monitoring and collection of specific outcome measures (22/32, 68%), the latter including patient-reported outcome measures (pain, fatigue, sleep, mood, and global well-being) and disease activity scores. Furthermore, many allowed self-visualization of the health data as a trend (17/22, 77%), such as disease activity scores and physical activity (measured by the number of steps). A few apps (7/32, 21%) aimed to promote physical activity through daily reminders and education on physical activity programs. For instance, 2 apps were designed to support coping mechanisms around pain management with relaxation therapy. Finally, 2 apps were designed primarily to help medication adherence through a tick-box option on the app when the medication is taken or through sending daily reminders with the possibility for the patient to edit the frequency of the reminders. None of the apps were reported as having the status of medical device.

Most of the apps addressed multiple disease features (detailed in Table 1).

**Table 1:** Features addressed by the different apps for rheumatoid arthritis and other rheumatic and musculoskeletal diseases.

Features addressed by the apps	Rheumatoid arthritis apps	Apps designed for other rheumatic and musculoskeletal diseases	
	Value, n (%)	Value, n (%)	Details on other diseases
Pain	7 (22)	13 (41)	JIA^a^, OA^b^, fibromylagia, PsA/AS^c^, and spine disease
Fatigue	4 (13)	9 (28)	JIA, OA, and fibromylagia
Physical activity	2 (6)	9 (28)	JIA and OA
Sleep	1 (3)	8 (25)	JIA, OA, and fibromylagia
Disease Activity Score	8 (25)	1 (3)	PsA/AS
Health Assessment Questionnaire	6 (19)	2 (6)	JIA and PsA/AS
Mood	0 (0)	6 (19)	JIA, OA, and fibromylagia
Global well-being (Short Form 36)	1 (3)	6 (19)	OA
Morning stiffness	1 (3)	4 (13)	JIA and PsA/AS
Depression/anxiety	1 (3)	2 (6)	Fibromyalgia
Medication/adherence	1 (3)	3 (9)	JIA, OA, and fibromylagia
Tender joint count	1 (3)	3 (9)	JIA
Gait	4 (13)	0 (0)	—^d^
Social support	0 (0)	2 (6)	JIA
Work	0 (0)	2 (6)	JIA
Grip	1 (3)	0 (0)	—

^a^JIA: juvenile idiopathic arthritis.

^b^OA: osteoarthritis.

^c^PsA/AS: psoriatic arthritis/ankylosing spondyloarthritis.

^d^Not applicable.

### Development Process of the Apps

The development process of the app was not described at all in 9% (3/32) of the studies (Table 2). Only 15% (5/32) of articles stated that patients were included in the development of the apps. A qualitative phase occurred in only 18% (6/32) of the cases [19,27,30,33,36,37]. This qualitative phase consisted of individual interviews (4 different studies), patient focus group (1 study), or patients focus groups and individual interviews (1 study). A mixed method approach was undertaken in 2 of those studies, with the addition of a patient survey or a Delphi procedure.

Health professionals and/or physicians were involved in the development or evaluation phase in 40% (13/32) of the studies.

**Table 2:** Description of the development phase and funding sources of the apps.

Reference	Quality of design and evaluation phase	Health care provider involvement	Patient involvement	Qualitative phase	Funding/development
Li et al, 2017 [16]	N/A^a^	N/A	N/A	N/A	Private
Skrepnik et al, 2017 [17]	B^b^	−^c^	+^d^	−	Private
Kristjansdottir et al, 2013 [18]	A^e^	−	+	+	Public
Kristjansdottir et al, 2013 [19]	A	−	+	+	Public
Paxton et al, 2018 [20]	B	−	+	−	Private
Harmelink et al, 2017 [21]	B	−	+	−	Private
Bults et al, 2010 [22]	B	−	+	−	Public
Kvien et al, 2005 [23]	B	−	+	−	Public
Heiberg et al, 2007 [24]	B	−	+	−	Not reported
Richter et al, 2008 [25]	B	+	+	−	Public
Stinson et al, 2008 [26]	A	+	+	+	Public
Stinson et al, 2006 [27]	A	+	+	+	Public
Garcia-Palacios et al, 2014 [28]	B	+	+	−	Public
Nishiguchi et al, 2016 [29]	B	−	+	−	Public
de la Vega et al, 2018 [30]	A	+	+	+	Public
Salaffi et al, 2013 [31]	B	−	+	−	Private
Yen et al, 2016 [32]	B	+	+	−	Public
Khurana et al, 2016 [33]	A	−	+	+	Private
Kim et al, 2016 [34]	B	−	+	−	Public
Cai et al, 2017 [35]	A	+	+	+	Public
Revenas et al, 2015 [36]	A	+	+	+	Public and private
Synnott et al, 2015 [37]	B	+	+	−	Public
Bromberg et al, 2016 [38]	B	−	+	−	Public
Bromberg et al, 2014 [39]	B	−	+	−	Public
Okifuji et al, 2011 [40]	B	−	+	−	Private
Nishiguchi et al, 2014 [41]	B	+	+	−	Public
Shinohara et al, 2013 [42]	B	−	+	−	Not reported
Walker et al, 2017 [43]	B	+	+	−	Private
Yamada et al, 2012 [44]	B	−	+	−	Public
Espinoza et al, 2016 [45]	N/A	N/A	N/A	N/A	Private
Kim et al, 2016 [46]	B	+	+	−	Public
Twiggs et al, 2018 [47]	N/A	N/A	N/A	N/A	Private

^a^Not applicable.

^b^Patients or health care providers involved in the evaluation but not in the design process.

^c^Absent.

^d^Present.

^e^Patients or health care providers involved in both design and evaluation phases.

### Evaluation of the Apps

Physicians were rarely involved in app evaluation (5/32, 15%). Patients were more frequently involved in app evaluation but mostly indirectly through their adherence to the app (12/32, 70%). A total of 17 apps proposed a direct evaluation through the use of a satisfaction scale (12/32, 70%) and/or an open or closed questionnaire (9/32, 52%). Satisfaction scores and comments were generally positive.

### Commercial Availability of the Apps

Moreover, 16 (50%, 16/32) articles included in this review were published between 2016 and 2017. Only a few apps described in the publications were commercially available (4/32, 12%) at the time of our web-based search; all were free of cost.

### Quality of the Available Apps

We performed quality score on the apps, which were existing in the different online stores (iTunes and Google Play) using a validated app quality score, the Mobile Application Rating Scale (MARS) [48]. The MARS includes different quality subscale scores, which rate engagement, functionality, aesthetics, and information. The score was calculated for the available apps. MARS for the ReaApp (Google Play) was 3.01 out of 5 and 3.8 out of 5 for the iGetBetter app (iTunes). The other apps were either not available online (28/32) or not in English language (1/32) or not accessible for free (1/32).

### Effectiveness of the Apps

A total of 2 RCTs were included in the SLR, 1 study in OA [17] and 1 in fibromyalgia [18,19]. In the trial by Skrepnik et al [17], patients were randomized in 2 groups: a mobile OA app along with a wearable activity monitor with (intervention group) or without feedback (control group). A significant increase in the number of steps per day (1199 vs 467, *P*=.03) as well as a reduction in pain from baseline during the 6-min walk test was shown in the intervention group. The trial by Kristjandottir et al [19] included a smartphone-delivered intervention with diaries and personalized feedback to patient living with fibromyalgia. The primary endpoint was met with a reduction of catastrophizing score in the treated group (mean 9.20, SD 5.85) compared with the control group (mean 15.71, SD 9.11; *P*<.001).

## Discussion

### Principal Findings

To our knowledge, this is the first piece of work to identify literature published on self-management mHealth apps for patients living with RMDs. Our search yielded heterogeneous studies, referring to heterogeneous apps designed either for a specific rheumatic disease or for multiple diseases and also for the general public/healthy population. The large quantity and variety of information that was collected by the app and the relevance of collecting this information as part of a self-management initiative are questionable and not always clearly outlined.

The development process of most apps has been insufficient or not described in the screened existing literature, which raises questions around their credibility. Importantly, most of the apps, despite being designed for patient use, involved neither patients nor health care providers in their development phase. These findings are in line with recently published work, showing that health care professionals were involved in only 35% (n=7) of the apps designed for RA patients [49]. This is a major concern as the absence of involvement of the relevant stakeholders might lead to inappropriate development tailored for the eventual user, including lack of assurance of content approval by specialists.

Our results highlight the unmet need for a standardization process to facilitate the convoluted and demanding processes required to develop mHealth apps. This matter goes beyond the scope of self-management apps for patients. For instance, Buijink et al [50] showed in a previous study that most mHealth apps designed for health care professionals were lacking authenticity details; authors, manufacturers, and distributors were not listed; and references were unavailable or out of date. Indeed, as mHealth apps are considered as medical devices by the US Food and Drug Administration, they should be subject to rigorous regulation [50]. These findings are consistent with our own findings and observations; indeed, the provenance and design process of the app as well as developers and funding sources are lacking details in many papers. Indeed, funding sources were not cited in more than half of the studies, which makes it difficult to identify clearly the nature of beneficiaries of such apps.

Moreover, despite more than half of the studies included in this review being published in the past 2 years, only a handful of apps were commercially available at the time of our search. This highlights the high turnover of apps developed for this purpose. One speculation could be that some of the apps were used in only 1 center and therefore have never been available for public use.

### Strengths and Limitations

It should be noted that our systematic review did not specifically address the question of reviewing apps existing in the Apple store. We focused on published literature on self-management mHealth apps, regardless of the type of RMDs. The very heterogeneous nature of the literature published on this topic and the relatively low number of relevant publications on the subject constitute the main limitations of this study. To ensure a most informative literature review, aside from systematically exploring the literature and other possible sources of information, we extracted data from the apps’ content and development procedure when it was provided. The latter revealed that many apps were usually focusing on selected aspects of disease self-management. Most were giving the patients the opportunity to enter selected PROs, especially fatigue, pain, and sleep, and also collected information on physical activity. Disease activity scores were more rarely (28.2%) collected by the apps. No relevant data were available on the quantitative use of apps.

Our work is in line with a recent study by Grainger et al who assessed the quality of RA apps specifically, highlighting the fact that of the 19 apps analyzed, only 1 had functionality to allow both the calculation of a validated composite disease activity measure and the ability to track calculated patient data [51]. Another recent work showed that most of the apps designed for patients living with RA did not offer a comprehensive experience. Comparably with what we found in the literature, not all apps (75%) offered a symptom tracking experience, and when it was the case, only a few apps allowed collecting PROs, joint counts, and laboratory results [49].

Thinking forward, a development process under specific guidelines or recommendations is mandatory to improve physicians’ as well as patients’ confidence in future apps. Having a meticulous development process in place can enhance appropriateness for the specific purpose they have been designed for, their scientific content and accuracy. By regulating the development process, the health care providers will also validate reliability of the scientific content and regulatory rules to ensure data protection and patient safety.

### Conclusions

In conclusion, despite patient willingness to use mHealth apps for self-management of their RMDs, better endeavors are needed to provide an optimal standard and ensure the quality and safety of new apps. This work will be used to further inform European League Against Rheumatism (EULAR) points to consider for development, evaluation, and implementation of mHealth apps for self-management of RMDs by patients. We hope through this work to stimulate some careful considerations around mHealth app development and evaluation, which will lead to a general effort to improve their value.

## Appendix

Multimedia Appendix 1 Search strategy for the hierarchical systematic literature review on mhealth Apps for disease self-management in patients with rheumatic and musculoskeletal diseases.

Multimedia Appendix 2 Venn Diagram demonstrating an example of article retrieval numbers using the largest database (Pubmed). N=435 represents the overlapping papers across three key domains searched.

Multimedia Appendix 3 Summary table of all 32 articles included in the review and type and purposes of studies included.

## Abbreviations

AS: ankylosing spondylitis
EULAR: European League Against Rheumatism
MARS: Mobile Application Rating Scale
mHealth: mobile health
OA: osteoarthritis
PICO: population, intervention, control, and outcomes
PROs: patient-reported outcomes
RA: rheumatoid arthritis
RCT: randomized controlled trial
RMDs: rheumatic and musculoskeletal diseases
SLR: systematic literature review
