# Supplementary material for: Mobile Health Apps for Self-Management of Rheumatic and Musculoskeletal Diseases: Systematic Literature Review
Source: JMIR Mhealth Uhealth. 2019 Nov 26;7(11):e14730. doi: 10.2196/14730 (PMC6904900; doi:10.2196/14730)
Supplement: Multimedia Appendix 1 [file mhealth_v7i11e14730_app1.pdf]

Online Supplementary Text S1.

Search strategy for the hierarchical systematic literature review on mhealth Apps for disease self-management in patients with rheumatic and musculoskeletal diseases. Search terms/strategy shown for each of the used databases.

*Ovid MEDLINE*

1. telehealth/exp
2. physical activity/exp
3. musculoskeletal disease/exp
4. mobile phone/exp AND [embase]/lim
5. mobile application/exp AND [embase]/lim
6. mobile device/exp AND [embase]/lim
7. mobile health/exp AND [embase]/lim
8. telemedicine/exp AND [embase]/lim
9. #1 OR #4 OR #5 OR #6 OR #7 OR #8
10. #2 AND #3 AND #9
11. #10 AND [embase]/lim NOT ([embase]/lim AND [medline]/lim)

*Embase*

#15. #3 AND #9 AND #14

#14. #12 OR #13

#13. ‘self-management’

#12. #10 OR #11

#11. personal AND (‘management’/exp OR management)

#10. ‘self-care’/exp AND [embase]/lim

#9. #4 OR #5 OR #6 OR #7 OR #8

#8. ‘telemedicine’/exp AND [embase]/lim

#7. ‘mobile health’/exp AND [embase]/lim

#6. ‘mobile device’/exp AND [embase]/lim

#5. ‘mobile application’/exp AND [embase]/lim

#4. ‘mobile phone’/exp AND [embase]/lim

#3. #2 OR #3

#2. ‘connective tissue disease’/exp AND [embase]/lim

#1. ‘musculoskeletal disease’/exp AND [embase]/lim

*The Cochrane Library*

("connective tissue disease":ti,ab,kw or "connective tissue disease" or "musculoskeletal disorder":ti,ab,kw or "musculoskeletal disorder" (Word variations have been searched)) AND ("telemedicine":ti,ab,kw or "telemedicine" or "mobile health":ti,ab,kw or "mobile health" (Word variations have been searched) or "mobile device":ti,ab,kw or "mobile device" or "mobile application":ti,ab,kw or "mobile application" (Word variations have been searched) or "mobile phone":ti,ab,kw or "mobile phone" (Word variations have been searched)) AND ("self care":ti,ab,kw or "self care" or "self collected":ti,ab,kw or "self collected" (Word variations have been searched));

*Psychinfo*

1. mobile Apps OR mobile Applications OR mobile device
2. self-management OR self-care OR self-rehabilitation OR self-regulation
3. (self-management OR self-care OR self-rehabilitation OR self-regulation) AND (S1 AND S2).
4. musculoskeletal diseases OR connective tissue disease
5. (musculoskeletal diseases OR connective tissue disease) AND (S3 AND S4)

*Web of Science*

#1. TS=(“mobile app”) OR TS=(“mobile applications”) OR TS=(mhealth) OR TS=(“mobile health”) OR TS=(“mobile apps”) OR TS=(“mobile application”) OR TS=(smartphone) OR TS=(“connected device”) OR TS=(“electronic device”) OR TS=(telemedicine) OR TS=(smartphones) OR TS=(pedometer)

#2. TOPIC: (musculoskeletal) OR TOPIC: (osteoarthritis) OR TOPIC: (“connective tissue disease”) OR TOPIC: (“rheumatic disease”) OR TOPIC: (lupus) OR TOPIC: (sjogren) OR TOPIC: (scleroderma) OR TOPIC: (“juvenile idiopathic arthritis”) OR TOPIC: (“adolescent arthritis polymyositis”) OR TOPIC: (dermatomyositis) OR TOPIC: (spondyloarthritis) OR TOPIC: (fibromylagia).

#3. TOPIC: (“self-management”) OR TOPIC: (“self-care”) OR TOPIC: (“patient reported outcome”)

#4. #3 AND #2 AND #1

*Pubmed*

*Step 1. SEARCH #1 related to Rheumatic and musculoskeletal diseases* (("musculoskeletal diseases"[MeSH Terms] OR ("musculoskeletal"[All Fields] AND "diseases"[All Fields]) OR "musculoskeletal diseases"[All Fields]) OR ("osteoarthritis"[MeSH Terms] OR "osteoarthritis"[All Fields]) OR ("connective tissue diseases"[MeSH Terms] OR ("connective"[All Fields] AND "tissue"[All Fields] AND "diseases"[All Fields]) OR "connective tissue diseases"[All Fields]) OR ("rheumatic diseases"[MeSH Terms] OR ("rheumatic"[All Fields] AND "diseases"[All Fields]) OR "rheumatic diseases"[All Fields]) OR ("lupus vulgaris"[MeSH Terms] OR ("lupus"[All Fields] AND "vulgaris"[All Fields]) OR "lupus vulgaris"[All Fields] OR "lupus"[All Fields]) OR sjorgen[All Fields] OR ("scleroderma, systemic"[MeSH Terms] OR ("scleroderma"[All Fields] AND "systemic"[All Fields]) OR "systemic scleroderma"[All Fields] OR "scleroderma"[All Fields] OR "scleroderma, localized"[MeSH Terms] OR ("scleroderma"[All Fields] AND "localized"[All Fields]) OR "localized scleroderma"[All Fields]) OR ("arthritis, juvenile"[MeSH Terms] OR ("arthritis"[All Fields] AND "juvenile"[All Fields]) OR "juvenile arthritis"[All Fields] OR ("juvenile"[All Fields] AND "arthritis"[All Fields])) OR ("polymyositis"[MeSH Terms] OR "polymyositis"[All Fields]) OR ("dermatomyositis"[MeSH Terms] OR "dermatomyositis"[All Fields]) OR ("spondylarthritis"[MeSH Terms] OR "spondyloarthritis"[All Fields]) OR ("fibromyalgia"[MeSH Terms] OR "fibromyalgia"[All Fields]))

*Step 2. SEARCH #2.* (("telemedicine"[MeSH Terms] OR "telemedicine"[All Fields] OR "mhealth"[All Fields]) OR m-health[All Fields] OR ("telemedicine"[MeSH Terms] OR "telemedicine"[All Fields]) OR "mobile application"[All Fields] OR "mobile applications"[All Fields] OR "mobile apps"[All Fields] OR (smartphone[All Fields] OR smartphone'[All Fields] OR smartphone's[All Fields] OR smartphoneassisted[All Fields] OR smartphonebook[All Fields] OR smartphones[All Fields] OR smartphones'[All Fields] OR smartphones4water[All Fields] OR pedometer* [All Fields]))

*Step 3. SEARCH #3* (("organization and administration"[MeSH Terms] OR ("organization"[All Fields] AND "administration"[All Fields]) OR "organization and administration"[All Fields] OR "management"[All Fields] OR "disease management"[MeSH Terms] OR ("disease"[All Fields] AND "management"[All Fields]) OR "disease management"[All Fields]) OR care[All Fields] OR (patient reported outcome[All Fields] OR patient reported outcomes[All Fields]) OR (patient reported outcome measure[All Fields] OR patient reported outcome measurement[All Fields] OR patient reported outcome measurements[All Fields] OR patient reported outcome measures[All Fields]) OR ("pain"[MeSH Terms] OR "pain"[All Fields]) OR ("fatigue"[MeSH Terms] OR "fatigue"[All Fields]) OR ("infection"[MeSH Terms] OR "infection"[All Fields]) OR ("infection"[MeSH Terms] OR "infection"[All Fields] OR "infections"[All Fields]) OR (morning[All Fields] AND stiffness[All Fields]) OR ("affect"[MeSH Terms] OR "affect"[All Fields] OR "mood"[All Fields]) OR ("depressive disorder"[MeSH Terms] OR ("depressive"[All Fields] AND "disorder"[All Fields]) OR "depressive

disorder"[All Fields] OR "depression"[All Fields] OR "depression"[MeSH Terms]) OR workout[All Fields] OR "disease activity"[All Fields] OR (("disease"[MeSH Terms] OR "disease"[All Fields]) AND ("motor activity"[MeSH Terms] OR ("motor"[All Fields] AND "activity"[All Fields]) OR "motor activity"[All Fields] OR "activity"[All Fields])) OR ("pharmaceutical preparations"[MeSH Terms] OR ("pharmaceutical"[All Fields] AND "preparations"[All Fields]) OR "pharmaceutical preparations"[All Fields] OR "medication"[All Fields]) OR ("motor activity"[MeSH Terms] OR ("motor"[All Fields] AND "activity"[All Fields]) OR "motor activity"[All Fields] OR "activity"[All Fields])).

*Step 4. Merging search #1 AND #2 AND #3*

*Grey Literature*

*List of societies, websites and stores*

https://www.apple.com/fr/ios/app-store/

https://www.healthline.com/health/best-ra-apps

https://www.eular.org

https://www.rheumatology.org

https://rheum.ca/fr

https://rheumatology.org.au
