# Supplementary figures and images for: Mobile Health Apps for Self-Management of Rheumatic and Musculoskeletal Diseases: Systematic Literature Review
Source: JMIR Mhealth Uhealth. 2019 Nov 26;7(11):e14730. doi: 10.2196/14730 (PMC6904900; doi:10.2196/14730)

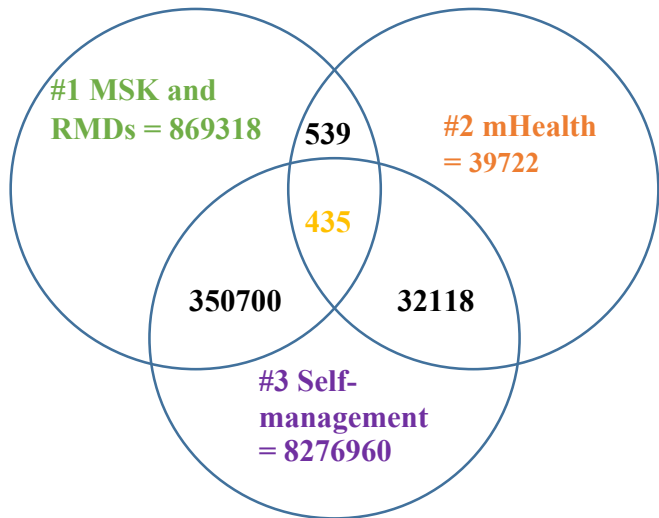

Supplement: Multimedia Appendix 2 [file mhealth_v7i11e14730_app2.pdf]
