# Supplementary material for: Mobile Health Apps for Self-Management of Rheumatic and Musculoskeletal Diseases: Systematic Literature Review
Source: JMIR Mhealth Uhealth. 2019 Nov 26;7(11):e14730. doi: 10.2196/14730 (PMC6904900; doi:10.2196/14730)
Supplement: Multimedia Appendix 3 [file mhealth_v7i11e14730_app3.pdf]

Supplementary Table 1. Summary table of all 32 articles included in the review and type and purposes of studies included.

First Author	Target Disease of the App	Country of Development of the App	App name	Data collected by the App	Purpose of each study
Li et al. [16]$	OA	US	FitBit Flex	Physical activity	Improvement of physical activity and mobility.
Skrepnik et al. [17]$	OA	US	OA GO	Sleep, Pain, Mood, Physical activity	Improvement of physical activity and mobility.
Kristjansdottir et al. [18]	Fibromyalgia	Norway	N/A	Fatigue, Sleep, Pain, Mood, Depression/Anxiety, Physical activity, Global Well being	Efficacy in reducing catastrophism
Kristjansdottir et al. [19]	Fibromyalgia	Norway	N/A	Fatigue, Sleep, Pain, Mood, Depression/Anxiety, Physical activity, Global Well being	Efficacy in reducing catastrophism
**Observational studies**					
Paxton et al. [20]$	OA	US	Fitbit Zip[R]	Sleep, Pain, Physical activity	Feasibility and efficacy of a self-rehabilitation program after TKA.
Harmelink et al. [21]$	OA	The Netherlands	PROMOVE3D	Physical activity	Feasibility and efficacy of a self-rehabilitation program after TKA.
Bults et al. [22]	Fibromyalgia	The Netherlands	N/A	Pain	Efficacy in reducing pain.
Kvien et al. [23]	RA	Norway	N/A	Fatigue, Pain, Disease activity, HAQ Well-Being, SF-36	Compare PROs and e-PROs
Heiberg et al. [24]	RA	Norway	N/A	Fatigue, Sleep, Pain, Disease activity, HAQ	Compare PROs and e-PROs
Richter et al. [25]	RA, SPA, LES	Germany	DocuMed.rh	Disease activity, SF-36	Compare PROs and e-PROs
Stinson et al. [26]*	JIA	Canada	e-Ouch	Fatigue, Sleep, Pain, TJC, Mood, morning stiffness, Social support, Work.	Assessment of App usability and/or acceptability
Stinson et al. [27]*	JIA	Canada	e-Ouch	Fatigue, Sleep, Pain, TJC, Mood, morning stiffness, Social support, Work.	Assessment of App usability and/or acceptability

Garcia-Palacios et al. [28]*	Fibromyalgia	Spain	F-EMA	Fatigue, Sleep, Pain	Assessment of App usability and/or acceptability
Nishiguchi et al. [29] *	RA	Japan	N/A	Gait, Pain, Disease activity, HAQ	Assessment of App usability and/or acceptability.
de la Vega et al. [30]*	Fibromyalgia	Spain	Fibroline	Pain, Fatigue, Sleep, Mood, Depression/Anxiety, Medication/Adherence	Assessment of App usability and/or acceptability
Salaffi et al. [31]*	Axial spondylitis	Italy	SPEAMonitor	Pain, Morning stiffness, BASDAI, BASFI ,HAQ	Assessment of App usability and/or acceptability
Yen et al. [32] *	RA	US	READY	Pain, Fatigue, Disease activity, Depression/Anxiety, HAQ, Morning stiffness	Assessment of App usability and/or acceptability
Khurana et al. [33]*	OA	US	N/A	Fatigue, Medication/adherence	Assessment of App usability and/or acceptability
Kim et al. [34]*	Spine disease	Korea	N/A	Pain	Assessment of App usability and/or acceptability
Cai et al. [35]*	JIA	UK	JIApp	Fatigue, Pain, Physical activity, Medication Adherence	Assessment of App usability and/or acceptability
Revenas et al. [36]	RA	Sweden	tRAppen	Physical activity	Description of the development and implementation process of an App
Synnott et al. [37]	Ankle sprain	Norway	ReApp	Physical activity	Description of the development and implementation process of an App
Bromberg et al. [38]	JIA	US	N/A	Fatigue, Sleep, Pain.	Assessment of temporal relation between pain, fatigue and other disease related variables
Bromberg et al. [39]	JIA	US	N/A	Fatigue, Sleep, Pain.	Assessment of temporal relation between pain, fatigue and other disease related variables
Okifuji et al. [40]	Fibromyalgia	US	N/A	Fatigue, Sleep, Pain, depression/Anxiety	Assessment of temporal relation between pain, fatigue and other disease related variables.

Nishiguchi et al. [41]	RA	Japan	N/A	Pain, Disease activity, HAQ, Gait.	Measurement of RA disease activity and other disease related variables using an App
Shinohara et al. [42]	RA	Japan	N/A	Pain, TJC, SJC, Disease activity, HAQ,	Measurement of RA disease activity and other disease related variables using an App
Walker et al. [43]	RA	Switzerland	COmPASS II	Fatigue, Sleep, Pain	Measurement of RA disease activity and other disease related variables using an App
Yamada et al. [44]	RA	Japan	N/A	Gait	Gait assessment using an App
Espinoza et al. [45]	RA	US	N/A	Grip, Disease Activity	Measurement of RA disease activity and other disease related variables using an App connected to a manometer
Kim et al. [46]	OA	US	IGetBetter[R]	Physical activity	Assessment of physical activity through an APP + connected device
Twiggs et al. [47]	OA	US	Fitbit Flex[R]	Physical activity	Assessment of physical activity through an APP + connected device

OA: Osteoarthritis, RA: Rheumatoid arthritis, JIA: Juvenile idiopathic arthritis, TJC: Tender Joint Count, SJC: Swollen joint count, HAQ: Health assessment questionnaire, BASDAI: Bath ankylosing spondylitis disease activity, BASFI: Bath ankylosing spondylitis functional index

Articles with the sign $ refer to an intervention related to the behavior and articles with * refer to an intervention related to monitoring based on the Behavioral Change Technique Taxonomy from Michie et al [47].
